# Supplementary material for: Understanding displacement of onboard contingents in Navy amphibious ships
Source: PLoS One. 2025 Jan 2;20(1):e0316266. doi: 10.1371/journal.pone.0316266 (PMC11695017; doi:10.1371/journal.pone.0316266)
Supplement: S1 File — (PDF) [file pone.0316266.s001.pdf]

Supporting information

S1 Appendix. Normality tests

The Anderson-Darling test is a non-parametric one that compares the empirical cumulative distribution function (ECDF) of the sample data with the expected distribution assuming the data follows a normal distribution. If the observed difference is sufficiently large ($p_{value} < 0.05$), the null hypothesis of normality of the population will be rejected.

$$S = \frac{1}{N}\sum_{k=1}^{N}(2\,k-1)\left(\ln F(Y_k) + \ln\left(1 - F(Y_{N+1-k})\right)\right) \tag{1}$$

The test statistic is compared with the distribution of the test statistic to determine the p_{value}.

The Ryan-Joiner test assesses normality by calculating the correlation between the data and the normal scores of the data. If the correlation coefficient is close to 1, it is likely that the population is normal. The Ryan-Joiner test statistic evaluates the strength of this correlation. If it falls below the appropriate critical value ($p_{value} < 0.05$), the null hypothesis of normality of the population will be rejected. This test is similar to the Shapiro-Wilk normality test.

$$R_p = \frac{\sum_{i=1}^{N}\left(Y_i - \overline{Y}\right)b_i}{\sqrt{s^2(n-1)\sum_{i=1}^{N} b_i^2}}, \tag{2}$$

where Y_i are the ordered observations, b_i are the normal scores of the ordered data, and s is the variance of the sample.

S2 Appendix. Independence tests

The Durbin-Watson statistic is defined as follows:

$$DW = \frac{\sum_{i=2}^{N}\left(E_i - E_{i-1}\right)^2}{\sum_{i=1}^{N} E_i^2}, \tag{3}$$

where $E_i = Y_i - Y_i'$, with Y_i and Y_i' as the real and estimated value, respectively.

To meet the assumption proposed by the Durbin-Watson statistic [1], assuming that the obtained errors are independent, this statistic should fall within the range of $[1.5, \ldots, 2.5]$.

Homoscedasticity or Levene's Test: It is an inferential statistical test that requires 3 or more groups or levels of factors. The null hypothesis assumes that the population variances are equal (homogeneity of variance or homoscedasticity). If $p_{value} < 0.05$, the

test is rejected, and it is concluded that there is a difference in variations within the population (heteroscedasticity):

$$W = \frac{(N-k)\sum_{i=1}^{k} N_i \left(Z_{i\cdot} - Z_{\cdot\cdot}\right)^2}{(k-1)\sum_{i=1}^{k}\sum_{j=1}^{N_i} \left(Z_{ij} - Zi\cdot\right)^2}, \tag{4}$$

where k is the number of different groups to which the sampled cases belong, N is the total number of cases across all groups, N_i is the number of cases in the i-th group, Z_{ij} is the value of the measured variable $(Y_{ij} - Y_i')$ for the j-th case in the i-th group, Y_i' is the mean of the i-th group, $Z_{\cdot\cdot} = \frac{1}{N}\sum_{i=1}^{k}\sum_{j=1}^{N_i} Z_{ij}$, and $Z_{i\cdot} = \frac{1}{N_i}\sum_{j=1}^{N_i} Z_{ij}$.

S3 Appendix. ANOVA

The Welch's t-test is an adaptation of Student's t-test with a 95% confidence level, which aims to validate the hypothesis that two populations have equal means. This test is applicable when the samples have different variances and sizes but still follow a normal distribution:

$$t = \frac{\bar{X}_1 - \bar{X}_2}{SE(\bar{X}_1 - \bar{X}_2)} \tag{5}$$

The Welch statistic is similar to the Student's statistic, except for the difference in calculating the standard error and degrees of freedom.

References

1. White KJ. The Durbin-Watson test for autocorrelation in nonlinear models. The Review of Economics and Statistics. 1992; p. 370–373.
