# Supplementary material for: Understanding displacement of onboard contingents in Navy amphibious ships
Source: PLoS One. 2025 Jan 2;20(1):e0316266. doi: 10.1371/journal.pone.0316266 (PMC11695017; doi:10.1371/journal.pone.0316266)
Supplement: Attachment — Submitted filename: Response to reviewers.docx [file pone.0316266.s002.docx]

PONE-D-23-34444

Dear Editors of PLOS ONE,

We would like to express our gratitude to the reviewers for the careful attention paid to this paper and their valuable remarks. Their comments are fully considered in the new version of the paper entitled “Understanding displacement of onboard contingents in Navy amphibious ships”', which is submitted again for your consideration. To this respect we have included several modifications aimed at clarifying the recommendations and comments pointed out by the reviewers. Additionally, we have also addressed all issues indicated by the Editorial board.

In our humble opinion, the paper may be valuable for the PLOS ONE journal audience. Moreover, In the following paragraphs we address all the comments made by the reviewers and describe the modifications introduced in the paper highlighted in yellow.

On behalf of the authors,

Rafael Asorey-Cacheda

**Response to editors:**

3. Thank you for stating the following financial disclosure: [This work was supported by the grants PID2020-116329GB-C22 and TED2021-129336B-I00 funded by MCIN/AEI/10.13039/501100011033 and by the European Union NextGenerationEU/PRTR.].

Please state what role the funders took in the study. If the funders had no role, please state: "The funders had no role in study design, data collection and analysis, decision to publish, or preparation of the manuscript." If this statement is not correct you must amend it as needed. Please include this amended Role of Funder statement in your cover letter; we will change the online submission form on your behalf.

**Due to the time that has passed since the paper submission, the financial disclosure has changed. Additionally, as any of funder had any role, we have added "The funders had no role in study design, data collection and analysis, decision to publish, or preparation of the manuscript." to the financial information:**

***This work was a result of the ThinkInAzul and AgroAlNext programmes, funded by Ministerio de Ciencia, Innovación y Universidades (MICIU) with funding from European Union NextGenerationEU/PRTR-C17.I1 and by Fundación Séneca with funding from Comunidad Autónoma Región de Murcia (CARM). This work was also supported by the grants PID2023-148214OB-C21 and TED2021-129336B-I00, funded by MICIU/AEI/10.13039/501100011033 and by the European Union NextGenerationEU/PRTR. This work was also funded by Fundación Séneca (22236/PDC/23). This research was also contextualized to DAIMon, a cascade funding action deriving from the Horizon Europe project aerOS, funded by the European Commission under grant number 101069732. The funders had no role in study design, data collection and analysis, decision to publish, or preparation of the manuscript.***

*4. Please expand the acronym “AEI” (as indicated in your financial disclosure) so that it states the name of your funders in full. This information should be included in your cover letter; we will change the online submission form on your behalf.*

**AEI states for “Agencia Estatal de Investigación”. The reason of only using the acronym is because it is how it is stated by AEI**

5. Thank you for stating the following in the Acknowledgments Section of your manuscript: This work was supported by the grants PID2020-116329GB-C22 and TED2021-129336B-I00 funded by MCIN/AEI/10.13039/501100011033 and by the European Union NextGenerationEU/PRTR] We note that you have provided funding information that is not currently declared in your Funding Statement. However, funding information should not appear in the Acknowledgments section or other areas of your manuscript. We will only publish funding information present in the Funding Statement section of the online submission form. Please remove any funding-related text from the manuscript and let us know how you would like to update your Funding Statement. Currently, your Funding Statement reads as follows: [This work was supported by the grants PID2020-116329GB-C22 and TED2021-129336B-I00 funded by MCIN/AEI/10.13039/501100011033 and by the European Union NextGenerationEU/PRTR.] Please include your amended statements within your cover letter; we will change the online submission form on your behalf.

This is the funding statement we want to be included:

***This work was a result of the ThinkInAzul and AgroAlNext programmes, funded by Ministerio de Ciencia, Innovación y Universidades (MICIU) with funding from European Union NextGenerationEU/PRTR-C17.I1 and by Fundación Séneca with funding from Comunidad Autónoma Región de Murcia (CARM). This work was also supported by the grants PID2023-148214OB-C21 and TED2021-129336B-I00, funded by MICIU/AEI/10.13039/501100011033 and by the European Union NextGenerationEU/PRTR. This work was also funded by Fundación Séneca (22236/PDC/23). This research was also contextualized to DAIMon, a cascade funding action deriving from the Horizon Europe project aerOS, funded by the European Commission under grant number 101069732. The funders had no role in study design, data collection and analysis, decision to publish, or preparation of the manuscript.***

6. Thank you for stating the following in your Competing Interests section: [NO authors have competing interests]. Please complete your Competing Interests on the online submission form to state any Competing Interests. If you have no competing interests, please state "The authors have declared that no competing interests exist.", as detailed online in our guide for authors at [http://journals.plos.org/plosone/s/submit-now](https://urldefense.com/v3/__http:/journals.plos.org/plosone/s/submit-now__;!!D9dNQwwGXtA!TpoROC0STVLQOt1Mn8WKiC_mgIUSXN_zNCABiWXx2MGnI80YAz3BWxQi7tuDkNoAEvuavFUOT2C7nRm-qeKB$) This information should be included in your cover letter; we will change the online submission form on your behalf.

**Please, indicate the following in the online submission form:**

***NO authors have competing interests.***

7. Please upload a copy of Supporting Information Figure/Table/etc. S1 Appendix. Normality tests which you refer to in your text on page 13.

**The Appendix “Normality tests” does not use supporting information. This appendix is introduced to explain the calculation method for this type of test, which is one of those used for the study presented in this article.**

8. Please review your reference list to ensure that it is complete and correct. If you have cited papers that have been retracted, please include the rationale for doing so in the manuscript text, or remove these references and replace them with relevant current references. Any changes to the reference list should be mentioned in the rebuttal letter that accompanies your revised manuscript. If you need to cite a retracted article, indicate the article’s retracted status in the References list and also include a citation and full reference for the retraction notice.

**The reference list has been checked. Regarding retracted paper, we have not found any in the reference list.**

**Response to reviewers:**

*3. Have the authors made all data underlying the findings in their manuscript fully available?

The* [*PLOS Data policy*](https://urldefense.com/v3/__http:/track.editorialmanager.com/CL0/http:*2F*2Fwww.plosone.org*2Fstatic*2Fpolicies.action*23sharing/1/010f019320d492a0-3afaf766-d803-4307-a42e-fa2abffacf13-000000/-s9q4bEU7me0Wcdg8uRdwpr-HX0Tz-ZHAYhU1oTM2M8=185__;JSUlJSU!!D9dNQwwGXtA!TpoROC0STVLQOt1Mn8WKiC_mgIUSXN_zNCABiWXx2MGnI80YAz3BWxQi7tuDkNoAEvuavFUOT2C7ne32p5OG$) *requires authors to make all data underlying the findings described in their manuscript fully available without restriction, with rare exception (please refer to the Data Availability Statement in the manuscript PDF file). The data should be provided as part of the manuscript or its supporting information, or deposited to a public repository. For example, in addition to summary statistics, the data points behind means, medians and variance measures should be available. If there are restrictions on publicly sharing data—e.g. participant privacy or use of data from a third party—those must be specified.*

*Reviewer #1: Yes*

*Reviewer #2: No*

**Unfortunately, we cannot disclose the data sets used for this study. Since the data used to carry out this displacement study have been extracted from a range of tests carried out by military personnel on board a Spanish Navy ship, these data are the property of the General Staff of the Navy (EMA) and, ultimately, of the Spanish State. It is also possible to use such data for the performance of a duly motivated and justified research, being mandatory to obtain an official authorization that enables the collection and processing of such data. This authorization will be issued by the owner of the data, in this particular case, by the EMA.**

5. Review Comments to the Author

*Please use the space provided to explain your answers to the questions above. You may also include additional comments for the author, including concerns about dual publication, research ethics, or publication ethics. (Please upload your review as an attachment if it exceeds 20,000 characters)*

*Reviewer #1: Excellent summary of a complex and important study. The supporting documentation and detailed method section with additional images and tables provide background to the project. There is nothing I would change, but more background and more literature review for those not as versed in this area may help.*

**Thanks for your comments. We do really appreciate them.**

*Reviewer #2: The manuscript is well written and I have some minor suggestions before the manuscript is good to go.*

*1. Please make the underlying dataset available - this is PLOS ONE's policy unless there is an justification (e.g., privacy, national security information) for not releasing the dataset publicly.*

***Since the data used to carry out this displacement study have been extracted from a range of tests carried out by military personnel on board a Spanish Navy ship, these data are the property of the General Staff of the Navy (EMA) and, ultimately, of the Spanish State. It is also possible to use such data for the performance of a duly motivated and justified research, being mandatory to obtain an official authorization that enables the collection and processing of such data. This authorization will be issued by the owner of the data, in this particular case, by the EMA.***

*2. Line 52, "Finally, Section “Conclusions” concludes", this phrase seems a bit weird. Can you rephrase it?*

**The phrase has been rewritten as:**

***Finally, Section “Conclusions” provides the closing remarks.***

*3. Have you got an ethical approval or at least an informed consent? PLOS ONE is very strict about this. Do mention it in your methodology or refer to the guidelines for authors.*

**Yes, all study participants were military personnel and provided informed consent. Moreover, we have modified the fourth paragraph of Section “Material and methods”, line 70, page 2, as follows:**

***To conduct the range of tests, the Spanish Navy was asked to designate the amphibious assault ship “Galicia” (L-51) as the study vessel during the course of regular national maneuvers in the Gulf of Cadiz and all participating military personnel provided informed consent. This type of ship was used since the platform's and its personnel's characteristics needed to match the planned research. This would be challenging due to the vessel's dimensions, the size of the reference population, and the heterogeneity of the personnel on board.***

*4. Can you provide a descriptive statistics of your research participants' demographics , such as age, gender, BMI, or seniority, etc.*

**This information is provided throughout the manuscript. Tables 1 and 2 describe the relevant demographic characteristics of the control groups. Thus, 10 control groups with members belonging to the demographic groups described in Tables 1, 2, and 6 were used for the study. Tables 3, 4, 5, 7, 8 and 9 show the values obtained for each of these groups (y0 to y9), while each row represents an individual belonging to one of the demographic groups. In this sense, to facilitate the interpretation of these tables (3, 4, 5, 7, 8, 9), we have chosen to add a new column on the left with the ID corresponding to the demographic factors in tables 1, 2 and 6.**

*5. Can you be more specific about the policy implications? Perhaps you can suggest what policy should relevant legal authorities should enact in light of your results (One paragraph).*

**Thanks for the suggestion. We have added the following paragraph at the end of the Section “Conclusions” (page 14, line 363):**

***Considering the study's findings, it is recommended that relevant jurisdictions adopt policies that promote data-driven approaches to decision making. Thus, ship evacuation plans should take into consideration and adjust to the demographic characteristics of the embarked population. In particular, these authorities should mandate regular data collection and validation to ensure the accuracy and relevance of data sets used in policy planning and plan development.***

*Overall a good job. If you cannot address some of the comments, please let me know why.*

**Thanks for this comment. We think we have addressed all of your comments, but the one regarding data availability due to security constraints.**
